# Applicability of Smartphone for Dynamic Postural Stability Evaluation

**DOI:** 10.1155/2019/9753898

**Published:** 2019-03-20

**Authors:** Jacek Polechoński, Agnieszka Nawrocka, Piotr Wodarski, Rajmund Tomik

**Affiliations:** ^1^Faculty of Physical Education, The Jerzy Kukuczka Academy of Physical Education in Katowice, Katowice, Poland; ^2^Faculty of Biomedical Engineering, Silesian University of Technology in Gliwice, Gliwice, Poland

## Abstract

The purpose of this study was to determine the accuracy of smartphone's gyroscope for dynamic postural stability among young healthy adults. The research included convenience sample of 85 healthy adults—37 women (mean age 22.1±1.6, body height 167.2±7.0) and 48 men (mean age 22.4±1.7, body height 176.1±13.8). In order to assess the accuracy of stabilometric measurement recorded by mobile phone, the raw data obtained at the same time by Sigma Balance Platform and Smartphone (SP) were correlated. Two thirty-second trials with one-minute interval break were performed (first in the frontal plane and second in the sagittal plane). A total of 170 measurements of postural stability were recorded (85 in frontal and 85 in the sagittal plane). The following parameters were included: the path of the stabilogram (in the case of SP, angular path) and the variation of the swing (standard deviation of the horizontal deflection of the platform). The results have shown strong and significant relationship between body sway variability measured by Sigma platform and smartphone in frontal (r=0.997) and sagittal (r=0.990) plane. For the geometric center of the platform and angular path distances, the correlation coefficient was also statistically significant and high, considering both lateral (r=0.999) and anterior-posterior sway (r=0.981). Our research shows that smartphones with gyroscope have potential for accurate assessment of postural balance, as an alternative for expensive and specialized equipment.

## 1. Introduction

In the last few years, the dynamic development of mobile technology has made smartphones (SPs) extremely versatile devices. Most smartphones have various built-in sensors (for example, accelerometer, gyroscope, magnetometer, gravity sensor, cameras, global positioning system (GPS), and thermometer) that are capable of providing raw data with high precision and accuracy. Therefore, the modern smartphones can monitor heart rate, sleep apnea, and gait parameters and also can estimate the energy expenditure of various physical activities. Such technical capabilities make these devices useful in daily life as well as medicine, rehabilitation, sport, and recreation. With connectivity to the Internet they are also an important part of telemedicine healthcare systems [1–7]. SPs with built-in accelerometer and gyroscope sensors are suitable for use in biomechanical measurements. Some studies have confirmed the possibilities of using mobile devices for postural balance assessment [8–11].

Two forms of balance should be considered: static and dynamic. Static balance is the ability to maintain postural stability and orientation with center of mass over the base of support and body at rest. Dynamic balance is the ability to maintain postural stability and orientation with center of mass over the base of support while the body parts are in motion [12]. Postural stability can be measured in many different ways. The common methods of postural stability assessment are both subjective clinical tests and advanced biomechanical measurements [13–17]. However, the first ones are inaccurate and for the next ones, specialized and expensive equipment is required. Moreover, computerized software and qualified personnel are essential for proper use of professional equipment. These issues all result in limitation of biomechanical techniques in common postural stability assessment.

Therefore, there is a need to search for alternative, inexpensive, portable, and commercially available devices equipped with applications and appropriate sensors which can be potentially used for objective stability assessment. Due to recent technological advances, we hypothesized that such role can be performed by modern smartphones. The purpose of this study was to determine accuracy of smartphone's gyroscope for dynamic postural stability among young healthy adults.

## 2. Material and Methods

**Figure 1:**
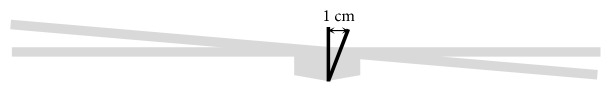
Range of movement of GCP Sigma.

The research included convenience sample of 85 healthy adults—students of Academy of Physical Education in Katowice: 37 women (mean age 22.1±1.6, body height 167.2±7.0) and 48 men (mean age 22.4±1.7, body height 176.1±13.8). Participants were asked to maintain the correct standing position with eyes open for 30 seconds on the Sigma Balance Platform (AC International East, software: Sigma Balance Platform v1.5242.24327, diameter: 42 cm, swing range: +15°/-15°, equipment compliant with Medical Devices Directive 93/42/EEC). The platform was set 1.5 meter from the white screen. The test was started by sound signal (beep). Two trials with one-minute interval break were performed (first in the frontal plane and second in the sagittal plane). A total of 170 measurements of postural stability were recorded (85 in frontal and 85 in sagittal plane). Before the measurements students were instructed to keep the balance platform perpendicular to the floor. Movements of the arms and trunk to help maintain balance were allowed. During the balancing the displacement of the geometric center of the platform (GCP) was recorded with 30 Hz sampling frequency. According to the Sigma Balance manual, the GCP range of motion is 1 centimeter and it results from the height of the platform and its maximum tilt (swing). Therefore, the maximum and minimum swing values of GCP are within 1 to -1 (Figure 1).

**Figure 2:**
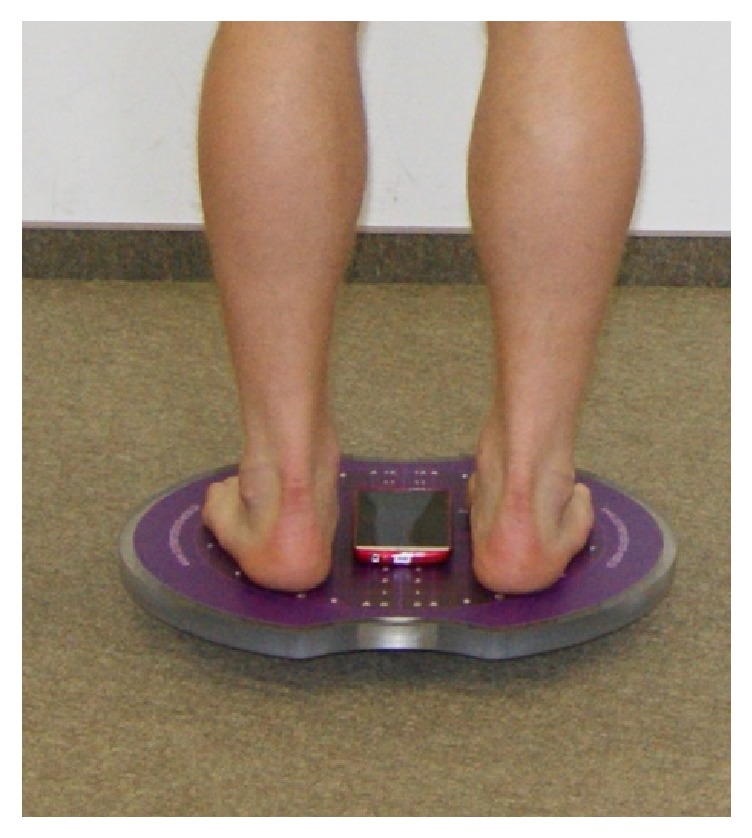
Location of the mobile phone on the Sigma Balance Platform.

The mobile phone (model LG G3) was fixed in the center of the Sigma Balance Platform. Therefore smartphone recorded data simultaneously with stabilometric platform (Figure 2).

For this purpose we used the SP's gyroscope forming part of the MPU-6500™ MotionTracking device made by InvenSense Inc. (version 1.0), which worked with Sensor Kinetics Pro app for Android phones (version 2.1.2) and recorded the angular displacements with 25 Hz sampling frequency. In order to assess the accuracy of stabilometric measurement recorded by mobile phone, the raw data obtained at the same time by Sigma and SP LG G3 were correlated. The following parameters were included: the path of the stabilogram (in the case of SP, angular path) and the variability of the swing (standard deviation of the horizontal deflection of the platform). Descriptive statistics were used to describe the basic features of the data: mean and standard deviations (SD). Pearson correlation was used to evaluate the relation between results achieved using Sigma Balance Platform and Smartphone. The Student t-test was also performed to assess the differences between postural balance of men and women. Analysis were performed using Statistica v.12 software (StatSoft Inc., USA).

## 3. Results

**Figure 3:**
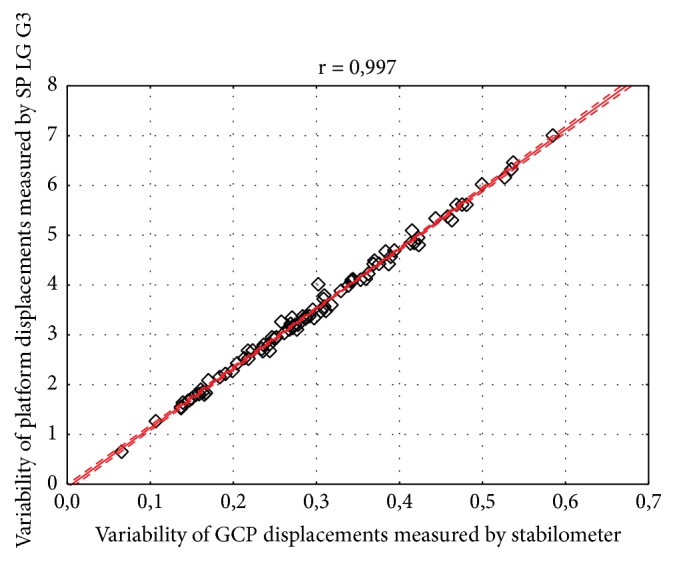
Correlation between body sway variability measured by Sigma Balance Platform and Smartphone (SP) LG G3 in the frontal plane. Legend: GCP: geometric center of the platform, r: correlation coefficient; solid line represents the linear regression with 95% confidence intervals indicated by the dashed lines.

**Figure 4:**
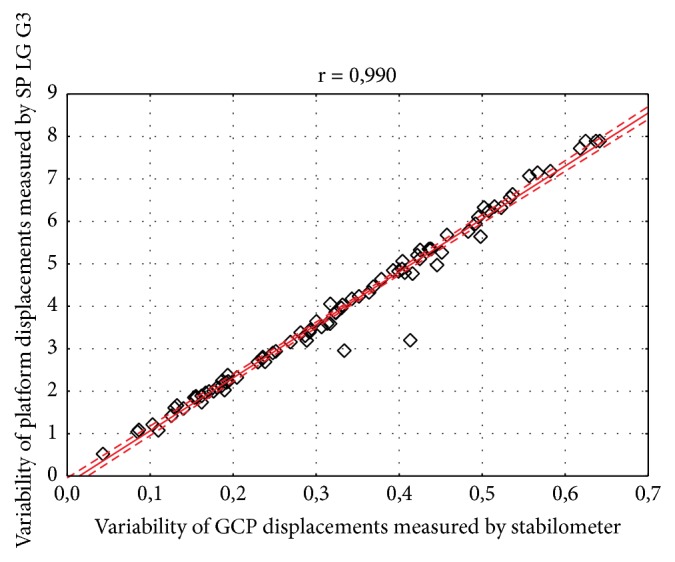
Correlation between body sway variability measured by Sigma Balance Platform and Smartphone (SP) LG G3 in the sagittal plane. Legend: GCP: geometric center of the platform, r: correlation coefficient; solid line represents the linear regression with 95% confidence intervals indicated by the dashed lines.

**Figure 5:**
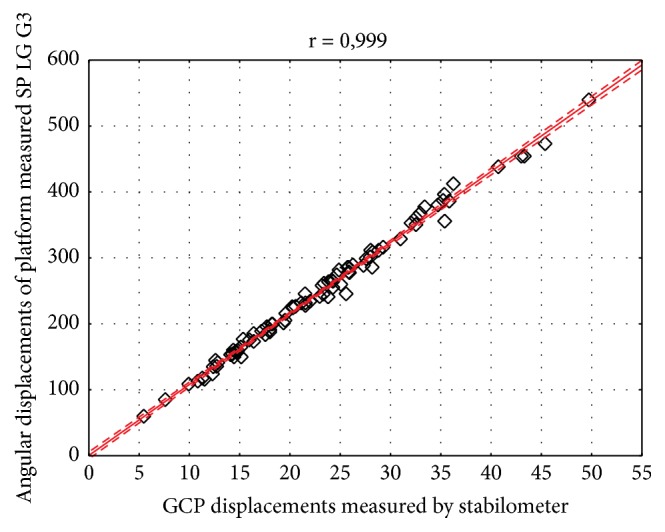
Correlation of the results obtained by Sigma Balance Platform (GCP path) and Smartphone (SP) LG G3 (angular path) in the frontal plane. Legend: GCP: geometric center of the platform, r: correlation coefficient; solid line represents the linear regression with 95% confidence intervals indicated by the dashed lines.

**Figure 6:**
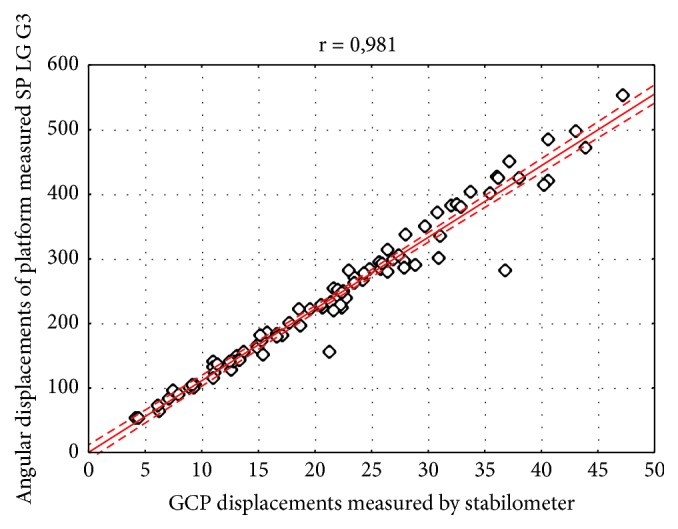
Correlation of the results obtained by Sigma Balance Platform (GCP path) and Smartphone (SP) LG G3 (angular path) in the sagittal plane. Legend: GCP: geometric center of the platform, r: correlation coefficient; solid line represents the linear regression with 95% confidence intervals indicated by the dashed lines.

The results have shown strong and significant relationship between body sway variability measured by Sigma platform and LG G3 smartphone in both frontal (r=0.997) and sagittal (r=0.990) plane (Figures 3 and 4). For the geometric center of the platform (GCP) and angular displacements the correlation coefficient was also statistically significant and high, considering both lateral (r = 0.999) and anterior-posterior sway (r = 0.981) (Figures 5 and 6).

By comparing the results of men and women, it appears that women presented better postural stability than men, regardless of the measurement device (Sigma platform and LG SP). Both GCP path measured by stabilometric platform and angular path measured by SP were shorter in the women, in comparison to the men. However, the significant differences were observed only in the sagittal plane. Similar relation was observed concerning the body sway variability, which was lower in all measurements of women, in comparison to men. However, only the results in anterior-posterior plane were statistical significant (Table 1).

## 4. Discussion

The purpose of this study was to determine accuracy of smartphone's gyroscope for dynamic postural stability assessment among young healthy adults. Our research has shown high and significant correlation between some stabilometric parameters measured by professional Sigma platform and gyroscope of smartphone in both frontal and sagittal plane. It confirms very dependable relationship between measurements obtained by both devices [18], which shows the possibility of using SP's gyroscope as accurate sensor for postural stability assessment.

In our research women in comparison to men achieved better postural stability parameters, independently of measuring device. This has also been observed by other authors [19–22]. This is probably caused by the fact that women have a slightly lower center of gravity than men.

So far, few studies have confirmed the potential and possibility of using mobile technology in assessing postural stability. However, they were performed using various devices, applications, and procedures. No research was found in which the smartphone located directly on the balance platform was used to record parameters as was the case in our measurements. Other authors placed them in various places on the subject's body.

Alberts et al. [8] tested the accuracy of consumer electronic device (iPad 2) with built-in accelerometer and gyroscope in postural stability assessment. They examined 22 men and 27 women, and they compared the data recorded simultaneously by iPad 2 placed on the height of sacrum and advanced postural system NeuroCom Balance Master during the Sensory Organization Test (SOT). The SOT is designed to systematically disrupt the sensory selection process by altering available somatosensory or visual information or both while measuring a subject's ability to minimize postural sway [23, 24]. According to the authors, the iPad 2 hardware provided data of sufficient precision and accuracy to quantify postural stability.

Other pilot research has been conducted by Patterson et al. [9]. They examined 13 men and 17 women, and they measured the postural sways using Biodex Balance System SD platform and Apple iPod Touch loaded with the Sway Balance Mobile Application software. Subjects were instructed to hold the iPod in an upright position, with the screen side of the device against the approximate mid-point of their sternum. Preliminary data showed strong consistency in the Sway Balance Mobile Application software outcomes when compared to those from the Biodex Balance System SD. According to the authors the results of their pilot studies are promising and confirm the potential of mobile technology in postural stability assessment. Balance measures, although with a small sample size, were consistent with measurements obtained using a previously validated system, demonstrating concurrent validity of the measurement using the Sway application on a handheld device.

Reliability of the Sway Balance Mobile Application was assessed by Amick et al. [10]. They studied 50 men and 9 women, and they performed the Sway Balance protocol twice per testing session over a period of three testing sessions. This protocol consisted of five stances including bipedal (feet together), tandem stance (left foot forward), tandem stance (right foot forward), single leg stance (right), and single leg stance (left). Each stance was performed on a firm surface with eyes closed for a period of 10 seconds, and each testing session was separated by a minimum of seven days. During measurements subjects pressed the mobile device to the sternum. Interclass Correlation Coefficients (ICC) were calculated as an indication of the test-retest reliability. Authors have shown that Sway Balance Mobile Application provides excellent overall reliability (ICC=0.76).

High reliability of measuring dynamic balance ability using a smartphone was shown by Han et al. [25]. The authors have examined 30 healthy young students in their 20s. The first and second rounds of the test were taken with one day interval to confirm the retest reliability. In each round, the dynamic balance ability was measured three times with eyes open and then another three times with eyes shut. Subjects stand on the Biodex Balance System with their bare feet put together and each of their hands holding their opposite shoulder. Balance was measured using a Galaxy Note 4 smartphone, with an Android 5.1.1v operating system and the application Kinetics Pro Sensor (version 2.1.2), the same as used in our research. Parameters of swings were recorded using an accelerometer and a gyroscope. During the measurement of the balance a smartphone was placed between the third and fourth lumbar vertebrae using Velcro and a plastic bag. The obtained values of ICC by accelerometer were 0.8 (eyes open) and 0.9 (eyes closed) in the measurements, and in the case of the gyroscope 0.7 (eyes open) and 0.6 (eyes closed). According to the authors, the results obtained in the research suggest that smartphones have limited potential for measuring equipment for dynamic balance ability.

Other interesting research has been conducted by Shah et al. [11]. They used three equal SPs devices with myAnkle application. The first device was allocated above the ankle, the second was allocated under knee, and the third was attached on the waist. Forty-eight participants completed 8 different balance exercises, separately for the right and left leg. Accelerometer data were obtained from each SP and mean acceleration was calculated for each exercise at each ankle and knee and the torso. In this research the authors concluded that myAnkle application is valid as compared with expert clinical ratings. According to Shah et al. [11] myAnkle application may have wider utility as a measurement tool for standing balance in clinical research and home settings.

Modern smartphones are already being used in many areas of daily life, such as communication, entertainment, education, and many others. Our research and observations indicated that smartphones with accelerometer and gyroscope can be also used for postural stability assessment as alternative to advanced and expensive monitors.

## 5. Limitations

When performing the tests with two devices, they are often calibrated and scaled to represent the same physical value. This method might help with the comparison of correlation between them and the assessment of the accuracy of the measurements. In the case of our research, conversion of angular values into linear values would be possible using trigonometric functions. However, such estimates could be fraught with error because of the lack of data on MEMS positions (microelectromechanical system) gyroscope in a cell phone (mainly height). Therefore, to avoid unnecessary approximations when importing values to the same units, the GCP displacements were performed.

## 6. Conclusions and Implication for Further Studies

To our knowledge, this is the first study that verifies correlation between assessment of postural stability using consumer smartphone gyroscope and professional balance platform in situation where smartphone was placed directly on the platform. Other authors placed them in various places on the subject's body.

Our research shows that smartphones with gyroscope have potential for accurate assessment of postural balance, as an alternative for expensive and specialized equipment. They could be used for a practical purpose by doctors, trainers, physiotherapists, teachers of physical education, and others. However, further studies are needed to assess the validity and reliability of postural balance measurement using smartphones, which will include large sample size with various ages and health conditions. Future research should also result in creation of simple application for mobile devices that will allow for fast interpreting and archiving of the postural balance data.

## Figures and Tables

**Table 1:** Comparison of dynamic balance parameters obtained by Sigma Balance Platform and smartphone among women and men.

Plane	Men (n=48)				Women (n=37)				d
	Mean	SD	Min	Max	Mean	SD	Min	Max	
Angular path measured by smartphone [rad]									

Frontal	264.09	95.48	108.60	539.13	230.60	98.39	59.91	454.12	33.49
Sagittal	273.45	122.35	53.07	552.49	207.89	98.62	63.13	450.16	**65.56** **∗**

GCP path measured by Sigma Balance Platform [mm]									

Frontal	24.52	8.79	9.95	49.76	21.33	9.15	5.47	43.03	3.19
Sagittal	24.46	10.89	4.15	47.20	18.91	8.69	6.08	40.17	**5.55** **∗**

Body sway variability measured by smartphone									

Frontal	3.62	1.13	1.64	6.46	3.36	1.60	0.65	7.00	0.27
Sagittal	4.35	1.82	0.52	7.89	3.39	1.89	1.04	7.89	**0.96** **∗**

Body sway variability measured by Sigma Balance Platform									

Frontal	0.31	0.09	0.14	0.54	0.28	0.13	0.07	0.58	0.02
Sagittal	0.36	0.14	0.04	0.64	0.28	0.15	0.08	0.64	**0.08** **∗**

GCP: geometric center of platform, *∗*p<0.05.

## Data Availability

The data used to support the findings of this study are available from the corresponding author upon request.
